# Health Effects of Long-Term Rapamycin Treatment: The Impact on Mouse Health of Enteric Rapamycin Treatment from Four Months of Age throughout Life

**DOI:** 10.1371/journal.pone.0126644

**Published:** 2015-05-15

**Authors:** Kathleen E. Fischer, Jonathan A. L. Gelfond, Vanessa Y. Soto, Chul Han, Shinichi Someya, Arlan Richardson, Steven N. Austad

**Affiliations:** 1 Barshop Institute for Longevity & Aging Studies, University of Texas Health Science Center San Antonio, San Antonio, Texas, United States of America; 2 Department of Physiology, University of Texas Health Science Center San Antonio, San Antonio, Texas, United States of America; 3 Department of Epidemiology & Biostatistics, University of Texas Health Science Center San Antonio, San Antonio, Texas, United States of America; 4 Departments of Aging and Geriatric Research, University of Florida, Gainesville, Florida, United States of America; 5 Department of Cellular & Structural Biology, University of Texas Health Science Center San Antonio, San Antonio, Texas, United States of America; Leibniz Institute for Age Research - Fritz Lipmann Institute (FLI), GERMANY

## Abstract

Rapamycin, an mTOR inhibitor, has been shown to extend lifespan in a range of model organisms. It has been reported to extend lifespan in multiple strains of mice, administered chronically or acutely early or late in life. The ability of rapamycin to extend health (healthspan) as opposed to life is less well documented. To assess the effects chronic rapamycin treatment on healthspan, enteric rapamycin was given to male and female C57BL/6J mice starting at 4 months of age and continued throughout life. Repeated, longitudinal assessments of health in individual animals were made starting at 16 months of age (=12 months of treatment) until death. A number of health parameters were improved (female grip strength, female body mass and reduced sleep fragmentation in both sexes), others showed no significant difference, while at least one (male rotarod performance) was negatively affected. Rapamycin treatment affected many measures of health in a highly sex-specific manner. While sex-specific phenotypic effects of rapamycin treatment have been widely reported, in this study we document sex differences in the *direction* of phenotypic change. Rapamycin-fed males and females were both significantly different from controls; however the differences were in the opposite direction in measures of body mass, percent fat and resting metabolic rate, a pattern not previously reported.

## Introduction

Rapamycin, a potent mTOR inhibitor, has been reported to extend lifespan in both vertebrate and invertebrate model organisms. In at least 7 previous studies mouse lifespan has been shown to be extended in both sexes, in heterogeneous and several inbred strains, with rapamycin administered in food (enteric rapamycin) or via injection, chronically or acutely, at a variety of ages [1–11]. However complete loss of mTOR signaling causes significant defects in growth and/or development in worms (*C*. *elegans*) and flies (*D*. *melanogaster*), and is embryonic lethal in mice [10]. The mTOR signaling network plays a pivotal role in promoting anabolic and inhibiting catabolic processes. mTOR activation stimulates ribosome biogenesis, protein synthesis and the import of nutrients into cells [12]; conversely, reduction or inhibition of mTOR activity decreases mRNA translation, increases autophagy, retards protein synthesis, slows cell growth and proliferation and enhances stress responsive transcription.

Because rapamycin treatment increases longevity across phylogenetically distant groups and is already FDA-approved for use in humans, it may represent a viable intervention to extend human life as well. Its effects on health have been less well documented. Recent evidence suggests that rapamycin may improve resistance to multiple, age-associated degenerative processes in mice. This offers the tantalizing possibility that rapamycin could extend the length of *healthy* human life. In mouse models rapamycin has been shown to delay the onset of Alzheimer’s pathology [13, 14], reduce the incidence of some cancers [4, 15–17], inhibit the development of atherosclerotic plaques [18], maintain cardiac function [1], enhance vaccine response in aged animals [5], delay age-related cognitive decline [19–21], and maintain some aspects of activity, motor function and behavior [1, 4, 11, 15, 16, 21–23]. On the other hand, rapamycin has been reported to have deleterious effects in mice, such as glucose intolerance and insulin resistance [24], testicular degeneration, increased cataract severity [15] and nephrotoxicity [21]. In some cases, results from different studies are inconsistent. For instance, some the beneficial effects on age-related changes found in one study (e.g. improved cardiac function with age [1] or increased insulin sensitivity [25]) have not been found in others (e.g. cardiac function [21], insulin sensitivity [24]). The potential use of rapamycin to address age-related diseases is promising, but the lack of consistent findings with respect to health in mice is reason for concern.

There are additional reasons for caution in considering rapamycin as a potential aging intervention. The use of rapamycin as part of immunosuppressive therapy after organ transplantation may be a reason for concern given age-related decline in immune function [26, 27]; however, recent research suggests that, in mice and primates, enterically delivered rapamycin may enhance rather than suppress some aspects of immune response (e.g. [28, 29]). Secondly, because it inhibits protein synthesis, cellular processes requiring *de novo* protein synthesis such as growth, tissue repair and regeneration may be compromised by chronic rapamycin administration. For example, some rodent studies have observed that mTOR inhibition retards recovery from skeletal [30] or cardiac muscle injury [31]. Additionally, rapamycin has been reported to negatively affect neuronal long-term potentiation and memory consolidation [32, 33]. Both human and rodent studies have associated inhibition of mTOR with insulin resistance [34]; however recent studies have suggested that these effects are transitory and diminish as duration of chronic treatment increases [1, 22].

Extending lifespan without delaying or diminishing age-related morbidity is not a desirable goal and rapamycin’s effects on healthspan were anything but clear. We therefore initiated a longitudinal study of rapamycin’s impact on longevity and a range of health parameters by treating C57BL/6 mice of both sexes with enteric rapamycin started at two distinct ages, 19 months (= old-fed or OF mice) and 4 months (= young-fed or YF mice) and continuing treatment throughout life. OF results have been previously published [11]. Consistent with our previous findings, survival of both males and females was modestly enhanced in YF animals [35]. Here we report on health impacts of enteric rapamycin treatment in YF mice of both sexes.

## Materials and Methods

### Animals and Husbandry

Three month old male and female C57BL/6J mice were purchased from the Jackson Laboratories. At four months of age 160 mice, 80 animals per sex, began receiving mouse chow (Purina 5LG6) containing either microencapsulated rapamycin (14 ppm) or empty capsules (eudragit = control), (n = 40 animals per sex and treatment group). Animals were maintained on their respective diets throughout life. Mice were assessed for blood levels of rapamycin at 10 months (i.e. after 6 months of treatment) and began assessments for a spectrum of health parameters starting at 16 months of age (after 12 months of treatment). All behavioral and physiological assays were performed in a single-blind design at regular intervals until an animal died or was euthanized for health reasons. Terminally ill animals were euthanized via continued exposure to CO2 for at least 15 minutes after respiratory arrest, followed by cervical dislocation. Unless otherwise indicated, assessments were performed during the dark (= active) phase of the 12:12 light cycle (grip strength, stride length, rotarod). Detailed descriptions of all experimental procedures may be found in our previous study of OF mice [11].

### Ethics Statement

Mice were maintained under barrier conditions by the UTHSCSA Nathan Shock Center Aging Animal and Longevity Assessment Core. All procedures for this study were approved by the Institutional Animal Care and Use Committee at the University of Texas Health Science Center at San Antonio. All animal died naturally or were euthanized for health reasons. All animals were closely monitored by project staff daily for health; animals that showed evidence of becoming ill were checked daily until the condition resolved or the animals was euthanized. Terminally ill animals were euthanized via continued exposure to CO2 for at least 15 minutes after respiratory arrest, followed by cervical dislocation.

### Rapamycin Quantification

Rapamycin concentration was quantified in uncoagulated whole mouse blood using high-performance-liquid chromatography (HPLC) with tandem mass spectrometry [11]. Blood was collected from both fasted and fed 10 month-old female and male mice (n = 17 and n = 14, respectively) that had been on rapamycin for 6 months.

### Food Consumption

Using a standard protocol [36], food consumption and body mass were measured in a subset of male and female, control and rapamycin-fed mice (n = 15 per sex and treatment group), starting at 5 months of age until 21 months of age.

### Body Composition

Fat-free mass, fat mass and free water were measured in all animals monthly, starting at 12 months of age (after 8 months of rapamycin treatment), using the EchoMRI quantitative nuclear magnetic resonance system (EchoMRI 3-in-1 System, Houston, TX).

### Energetics and Spontaneous Activity

Following a 12 hours acclimation period, oxygen consumption, carbon dioxide production, total metabolism and resting metabolic rate were measured over a 24-hour period using a MARS indirect calorimetry system (Sable Systems International, Las Vegas, NV). Spontaneous activity and its temporal patterns were assessed in individually-housed animals over twenty-four hours, one light and one dark phase, following a twelve hour acclimation period. Animals were placed in clear, Plexiglas, (40.6 x 22.9 x 14.0 cm) cages surrounded by a 2.5 cm grid of infrared sensors in the x and y plane with *ad lib* access to food and water.

### Movement and Strength Parameters

#### Gait

Senescence induces changes in the musculoskeletal system, including many pathophysiological conditions such as arthritis. Gait analysis provides a noninvasive method of assessing these changes. Gait parameters were measured using the TreadScan (Clever Sys, Reston, VA) apparatus. Mice were started at 12 cm/s and treadmill speed was adjusted until each mouse maintained a consistent walking speed for 5 minutes. TreadScan uses a high-speed digital camera to record reflected images of the mouse’s footpads at 80 frames per second and the images analyzed using mouse-specific algorithms to produce an assessment of more than 40 gait parameters. As many of these parameters are autocorrelated, we focused on stride length, which is likely to be affected by joint pain, neuromuscular deterioration and/or incipient kyphosis, as a reasonable parameter to reflect musculoskeletal health.

#### Rotarod

Mice were trained to use the rotarod during four sessions, spanning two weeks using the protocol described in our previous study [11]. Briefly, the mouse is balanced on the rod and the instrument started at 4rpm with an acceleration of 0.2 rotations/second. The maximum duration the mouse was able to balance on the rod (latency to fall) from six trials during the final fifth session was used to assess rotarod performance.

#### Grip strength

In humans, hand grip strength declines with increasing age and has been used widely as a component of frailty assessments [37]. We assessed mouse fore and hind-limb grip strength using a Grip Strength Meter with mesh grid pull bar (Columbus Instruments 1027 CSM). Mice grasped the pull bar with all four paws and were gently pulled backward in a horizontal plane until they released their grip. Maximum force during each pull (= 1 trial) is recorded and the highest value from 5 consecutive trials was used as the mouse’s maximum grip strength for that age.

### Inner Ear Histology

Dietary restriction slows the progression of age-related hearing loss in C57BL/6 mice as well as slowing the loss of spiral ganglion neurons and both outer and inner hair cells of the cochlea [38]. Because mTOR inhibition by rapamycin has been hypothesized to resemble dietary restriction [39], we examined cochlear histology at natural death in a subset of mice. Techniques were those of Someya [40]. Briefly, cochleae from control (males, n = 13; females n = 18) and rapamycin-fed (males, n = 5; females n = 12) animals were excised from formalin-preserved mice, decalcified in 10% ethylenediamintetracetic acid. Paraffin-embedded sections were stained with haematoxylin and eosin and examined under a light microscope. Rosenthal’s canal was divided into three cochlear regions: apical, middle and basal, and each region was evaluated separately. The number of spiral ganglion neurons was counted on digital photomicrographs of each canal profile and determined as the number of neurons per mm^2^. Percent survival of outer hair (OH) cells was calculated as the ratio intact OH cells present relative to the three OH cells normally observed in each turn of one cochlea in sections of mice with intact hearing. Inner hair (IH) cell survival was calculated as the number of intact IH cells compared to the number expected in each turn of the cochlea in tissues of mice with normal hearing.

### Statistical Methods

The continuous healthspan outcomes (body mass, body composition, metabolism, activity, etc.) were analyzed using linear random-effects models (with a random intercept) to account for the correlations between longitudinal measures of the same mouse. The main effects rapamycin treatment, sex, and age and their interactions were considered as potential predictors of healthspan. Because the effect of age on performance could be curvilinear (e.g., on body mass), we used polynomial transformations of age of up to order 3. We modeled males and females both together (for greater power to detect smaller effects consistent between males and females) and separately if, for example, there was a significant interaction between treatment and sex. For assays such as rotarod performance that are known to be affected by body size, we performed both body mass-adjusted and unadjusted analyses. The analytical computations were performed using R (v2+, Vienna, Austria).

## Results

### Rapamycin Blood Levels

**Fig 1:**
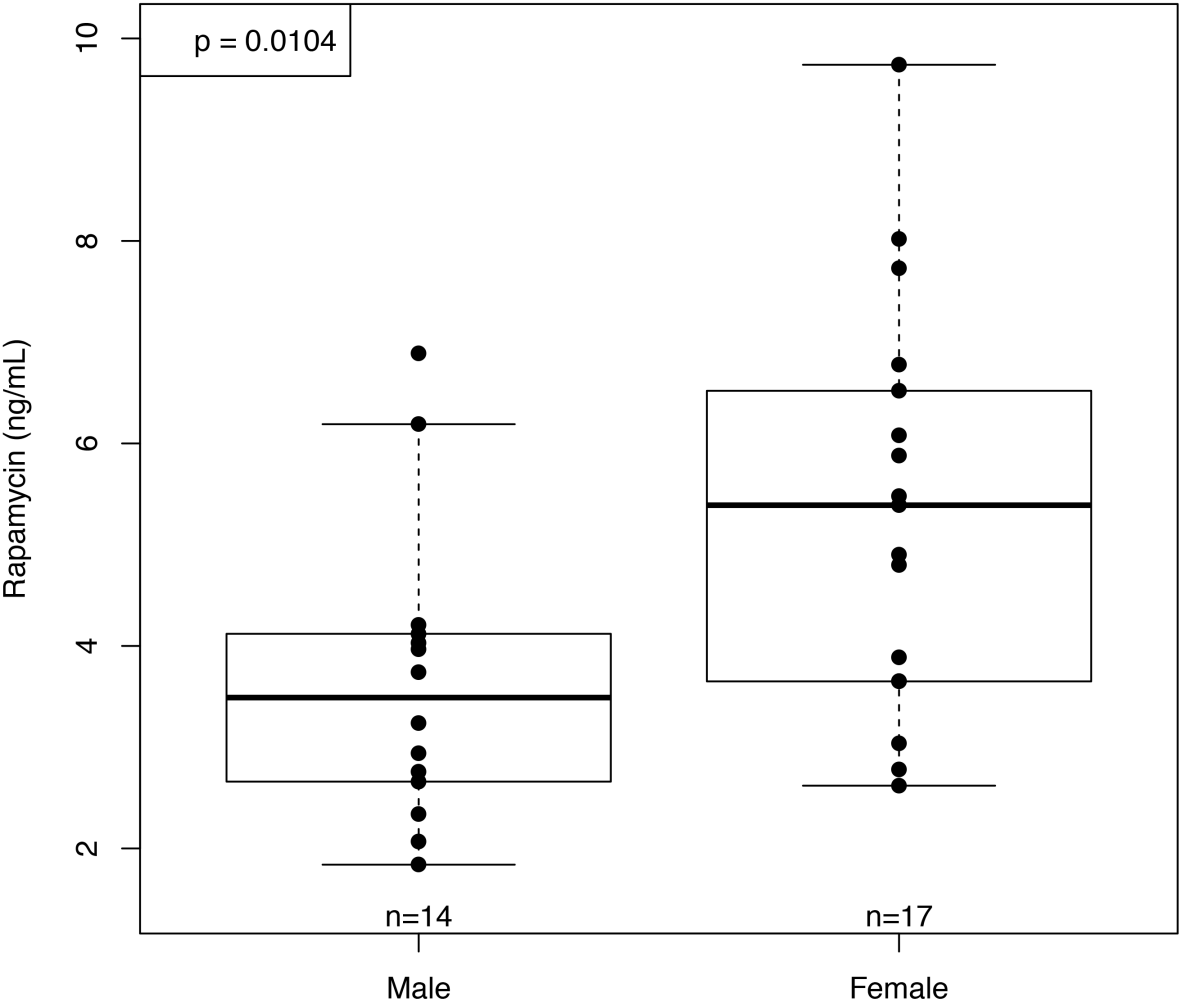
Rapamycin concentration in whole blood at 10 months of age (after 6 months of rapamycin feeding). Blood concentrations of rapamycin were significantly higher in females than males after 6 months of rapamycin feeding (n = 18 and 14 respectively).

Blood levels of rapamycin were comparable to those measured in our previous study of C57BL/6 receiving enteric rapamycin started at 19 months of age and measured 6 months later (old-fed mice, OF) [11]. Unlike our previous results in OF mice, the YF females showed significantly higher blood levels of rapamycin than YF males (Fig 1). The sex difference in rapamycin blood levels of YF mice was not due to differences in food consumption, as rapamycin-fed females did not consume more food per gram of body mass at testing age than did rapamycin-fed males (*p* = 0.83). This is in contrast to our observations in old-fed mice, where OF females consumed significantly more food per gram body mass than males (*p* = 0.004) but showed no differences in rapamycin levels [11]. These results are consistent with those obtained by Fok et al [35] in a companion study previously reported here. In this study, females also showed significantly higher blood levels of rapamycin than males at 10 months of age; however, rapamycin levels in liver tissue measured at 25 months of age were no different between males and females [35]. Fok et al reported that mTOR activity in the liver, as measured by the ratio of phosphorylated S6 Kinase 1 to total S6 Kinase 1, did not differ between control and rapamycin-fed animals at 21 months of age; however, mTOR transcripts in the liver were significantly increased in rapamycin-fed females and a subset of the rapamycin-fed males [35].

### Body Mass and Composition

**Fig 2:**
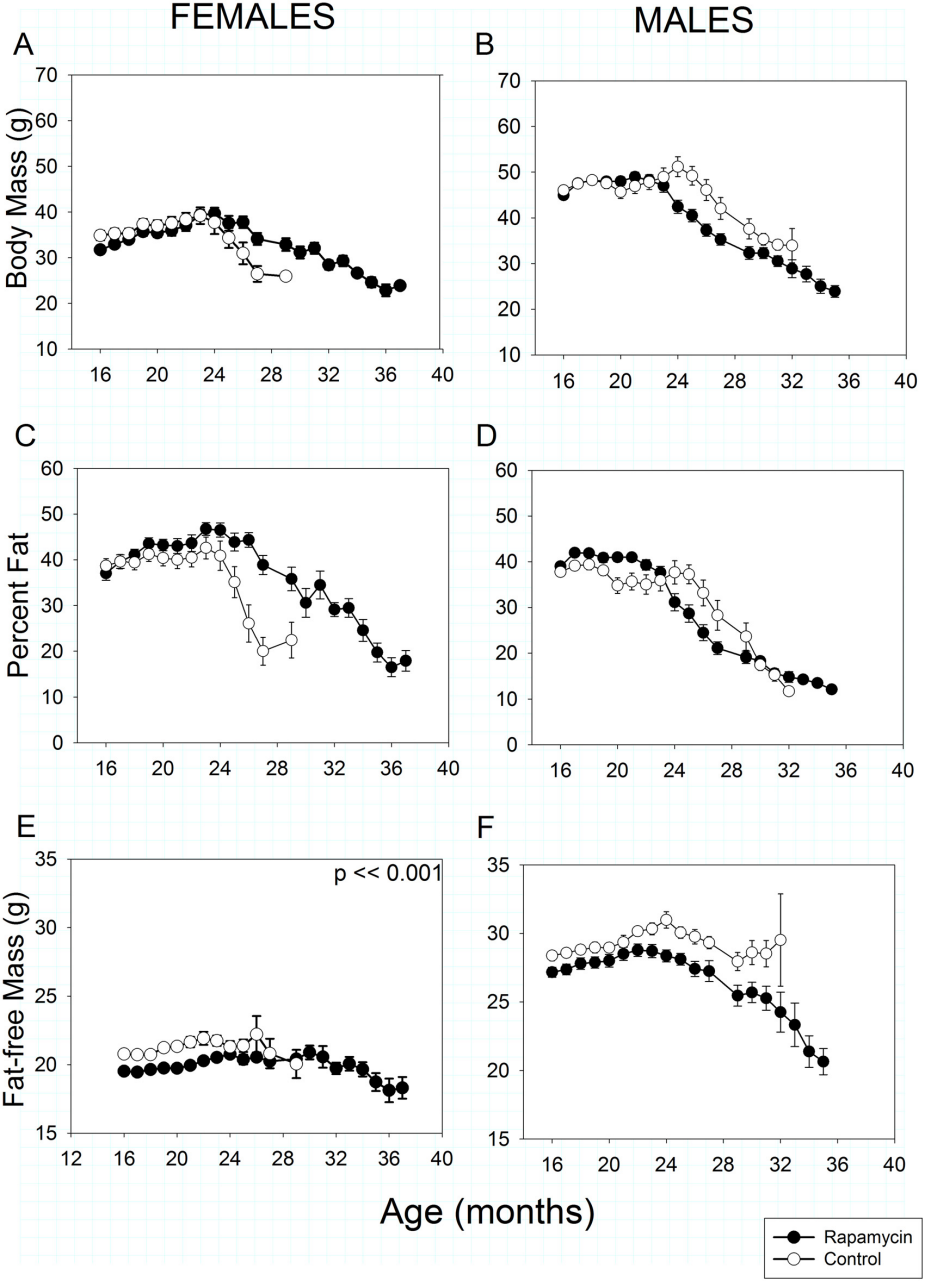
Body mass and composition in rapamycin-fed mice (filled circles) versus controls (hollow circles). P-values shown on individual panels only if there is a significant treatment effect independent of age. Sample sizes varied, depending on age, control females, n = 32–2; rapamycin females, n = 30–4; control males, n = 33–2; rapamycin males, n = 36–3. **A, B: Total body mass.** Highly significant differences (p << 0.001) exist in treatment x age effects in body for both sexes. Although they weighed less than controls by 16 months of age, rapamycin-fed females retained body mass longer, whereas rapamycin-fed males were similar to controls at 16 months but lost body mass earlier and remained lighter as they aged. **C, D: Percent body fat.** Highly significant differences (p << 0.001) in treatment x age effects exist for both sexes. As in with body mass, aging rapamycin-fed females retained body fat longer and lost body fat more slowly than age-matched controls. In contrast, rapamycin-fed males initially had a higher percentage of body fat, but lost fat mass earlier than controls. **E, F: Fat-free mass,** sometimes referred to as lean mass. Although obscured by the scaling, rapamycin-fed females had lower fat-free mass than controls at all ages measured. Fat-free mass declined more slowly with age in rapamycin-fed females than males.

In both sexes and treatments, total body mass and percent fat declined progressively beginning at 20–25 months of age (Fig 2A–2D). At 16 months of age, after one year of treatment, rapamycin-fed females weighed slightly less than controls (Treatment effect, *p* = 0.04) due to lower fat-free mass. However, once weight loss began rapamycin-fed females lost body and fat mass significantly more slowly with age than controls due to a slower loss of fat (Fig 2A and 2C: Treatment X Age interaction, *p* << 0.001 in both cases). Rapamycin treated females had less fat-free mass than controls at all ages measured (Treatment effect, *p* << 0.001, Fig 2E). Fat-free mass also declined significantly with age in both rapamycin-treated and control-fed females (Treatment X Age interaction, *p* < 0.001), although this loss is obscured somewhat by the scaling in Fig 2, which is designed to allow easy comparison between sexes. The degree of fat-free mass loss with age was substantially lower in rapamycin-fed females than males (Fig 2E vs 2F).

Control and rapamycin-fed males did not differ in body mass or composition at 16 months of age, but lost mass with aging significantly differently (Treatment X Age interaction, *p* < 0.001). Rapamycin-fed males began losing body mass about one month earlier than controls (Fig 2B), although once it began to decline, mean body mass in both groups fell at similar rates with age such that from about 25 months of age rapamycin-fed males consistently weighed less than controls. For both rapamycin-fed and control males, trajectories of age-related changes in both fat mass and fat-free mass were similar to those in total body mass.

### Metabolism and Spontaneous Activity

**Fig 3:**
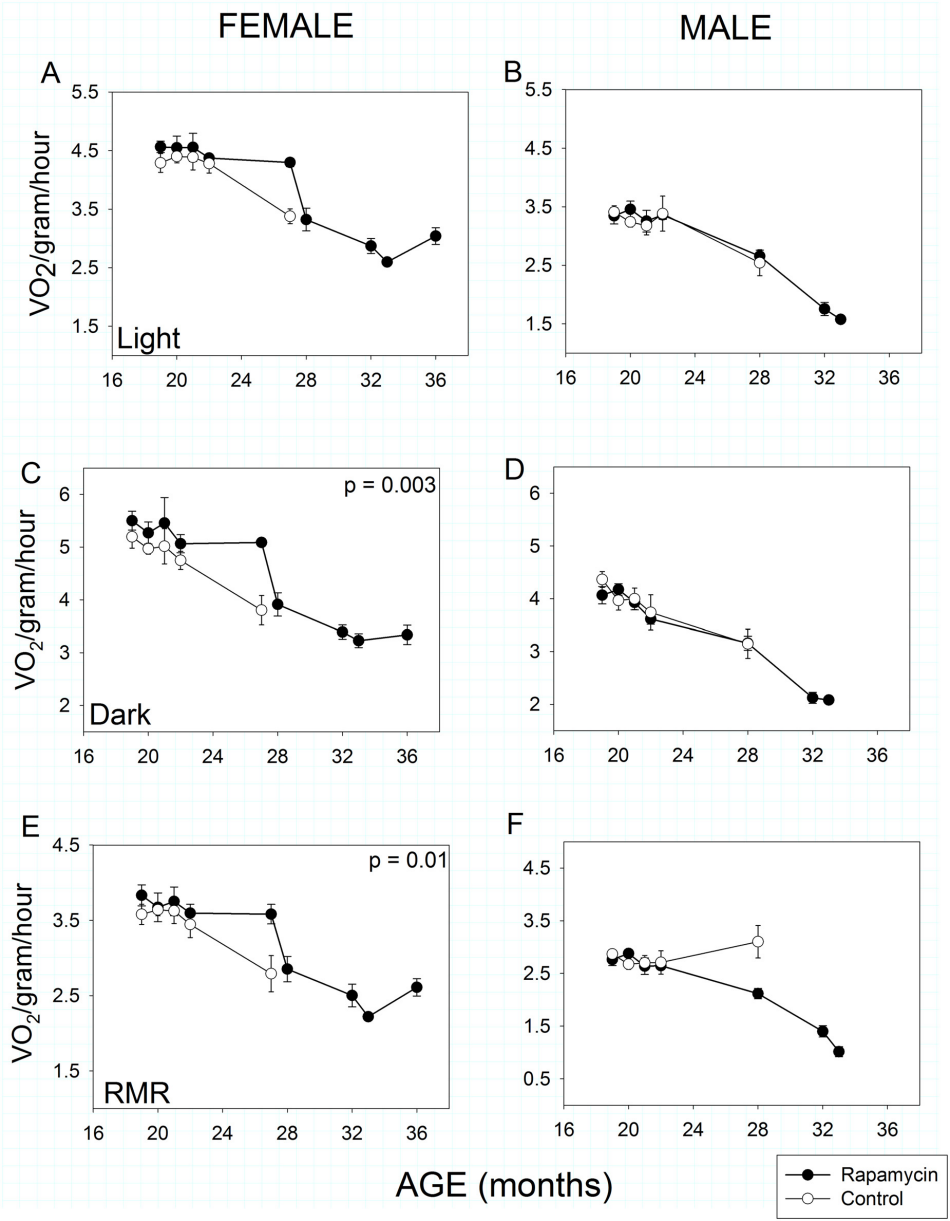
Metabolic activity in rapamycin-fed mice (filled circles) compared to controls (hollow circles). P-values shown on individual panels only if there is a significant treatment effect independent of age. Sample sizes varied, depending on age, control females, n = 8–4; rapamycin females, n = 11–3; control males, n = 9–5; rapamycin males, n = 16–2. **A, B: Mass-specific metabolic rate during the light (= inactive) phase.** Males and females showed no effects of rapamycin treatment on mass-specific metabolic rate during the inactive phase of their daily 24-hour cycle, although both sexes showed highly significant (p << 0.001) sex x age treatment effects. **C, D: Mass-specific metabolic rate during the dark (= active) phase.** Aging rapamycin-fed females, but not males, maintained significantly higher metabolic rates between measures taken at 24 and 28 months of age compared to controls during the dark (= active) phase of the 24-hour light cycle. Both males and females showed highly significant (p <<0.001) decline dark-phase metabolic rate with age irrespective of treatment. **E, F: Resting mass-specific metabolic rate.** Resting metabolic rate declined with age in females, but aging rapamycin-fed females had higher resting metabolic rates compared to age-matched controls. Resting metabolic rate declined significantly in aging rapamycin-fed males but not in age-matched controls (treatment x age, p << 0.001).

A meaningful interpretation of metabolism requires accounting for body mass and composition because fat-free mass is significantly more metabolically active than fat mass. Therefore, our metabolic rate measures are adjusted to amount of fat-free mass. According to this measure, all mice showed an age-related decline in total, mass-specific metabolic rate irrespective of sex or treatment from about 20 months of age (Age effect, *p* << 0.001 in all cases). In females, there was no significant effect of rapamycin treatment during the light (= inactive) phase of the 24 hour light:dark cycle (Fig 3A), but rapamycin-fed females exhibited higher *total* mass-specific metabolic rate during the dark (i.e., active) phase (Fig 3C: Treatment effect, *p* = 0.003) and a higher mass-specific *resting* metabolic rate (Fig 3E: Treatment effect, *p* = 0.01) irrespective of light:dark cycle.

By contrast, males showed no differences in total metabolic rate as a function of treatment in either phase of the 24 hour light:dark cycle. Interestingly, control males showed no decline in *resting* metabolic rate with age, whereas it declined significantly in the rapamycin-fed group (Fig 3F), thus there was a highly significant Treatment X Age interaction (*p <*< 0.001) in males.

**Fig 4:**
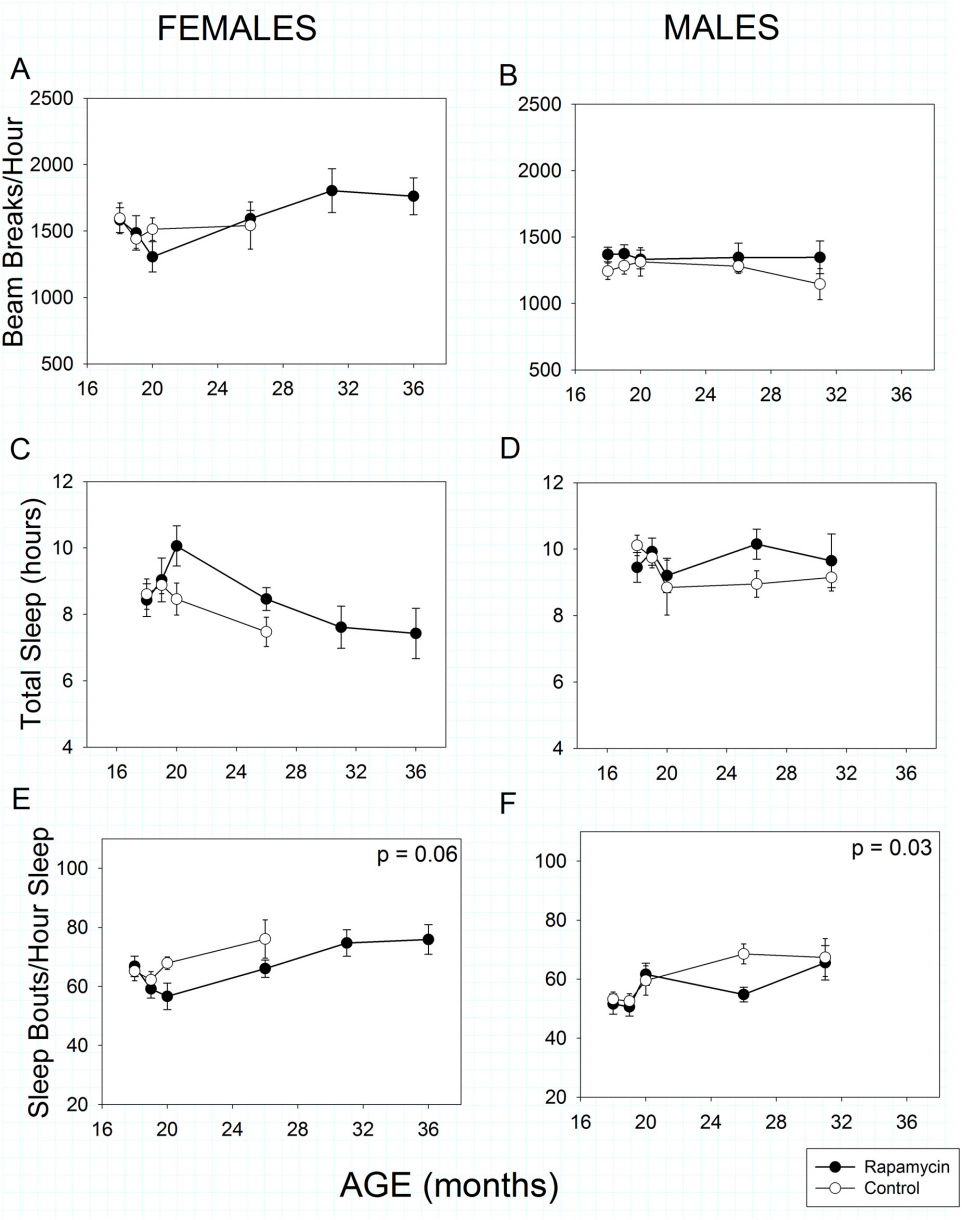
Spontaneous activity and sleep in rapamycin-fed mice (filled circles) compared to controls (hollow circles). P-values shown on individual panels only if there is a significant treatment effect independent of age. Sample sizes varied, depending on age, control females, n = 14–2; rapamycin females, n = 18–5; control males, n = 13–4; rapamycin males, n = 22–7. **A, B: Spontaneous 24-hour activity** was greater in females than males (p<< 0.001). Activity increased with age (p = 0.008) in females and decreased with age in males (p = 0.003), regardless of treatment. **C, D: Total sleep** Rapamycin treatment marginally increased total sleep in both sexes (p = 0.05) when taken together, but aging affected sleep patterns in the two sexes differently (age x sex, p << 0.001). **E, F: Sleep fragmentation** increased with age in all animals (p << 0.001); however, rapamycin treatment reduced sleep fragmentation in males and showed a trend to reduce it in females.

Total spontaneous activity over 24 hours was statistically greater in females than males (Fig 4A vs 4B, Sex effect, p<<0.001). Surprisingly, spontaneous activity as we measured it increased with age in females (Age effect, p = 0.008) but decreased as expected in males (Age effect, *p* = 0.003). Rapamycin feeding had no impact on total activity in females (Treatment effect, *p* = 0.54) or males (Treatment effect, *p* = 0.39).

Temporal patterns of sleep were monitored by analyzing bouts of inactivity greater than 40 seconds in length as previously validated for male C57BL/6 mice [11, 41]. Using this metric, females, but not males, slept less as they aged (Fig 4C, Age effect, *p* < <0.001). Rapamycin-feeding resulted in a marginally significant increase in total sleep time (Treatment effect, *p* = 0.05) when the sexes were combined, but not when each sex was treated separately (females: Treatment effect, *p* = 0.10, males: Treatment effect, *p* = 0.26). Sleep fragmentation, as assessed by the number of sleep bouts per hour of sleep, increased with age in all animals (Fig 4E and 4F, Age effect, *p* << 0.001) as has been previously reported for both humans and mice. However, consistent with our OF results, rapamycin treatment reduced sleep fragmentation in males relative to controls (Treatment effect, *p =* 0.03). Unlike our OF results, rapamycin treatment reduced sleep fragmentation marginally in females (Treatment effect, *p* = 0.06) compared to controls. Because this high-throughput sleep assessment protocol has only been validated in C57BL/6 males, the above results for females should be interpreted with caution.

### Strength and Movement

**Fig 5:**
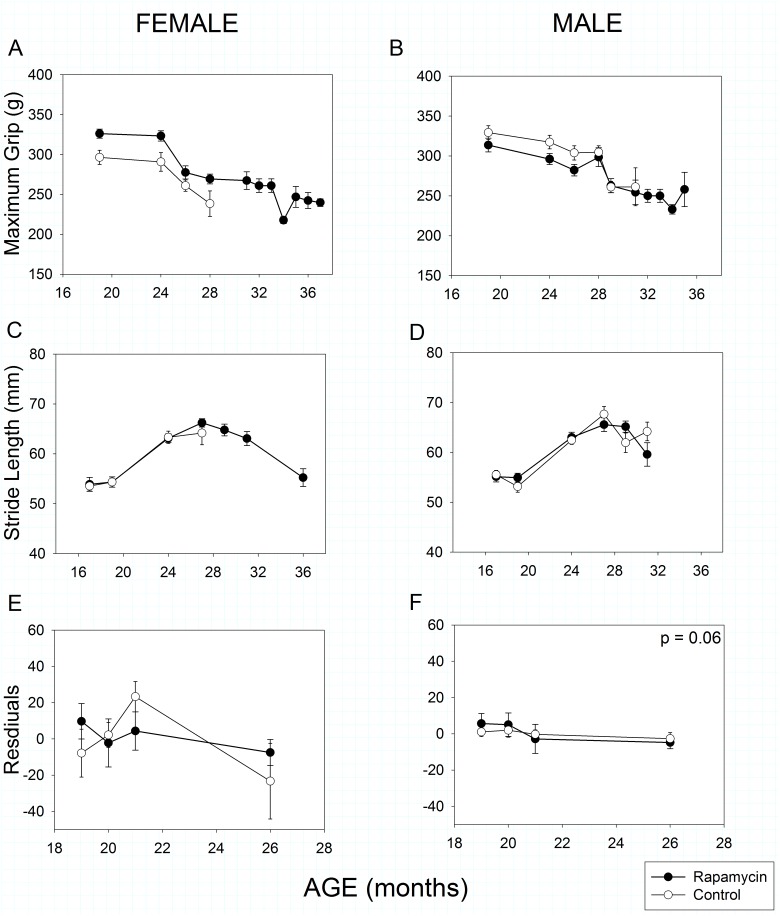
Strength, coordination and movement in rapamycin-fed mice (filled circles) compared to controls (hollow circles). P-values shown on individual panels only if there is a significant treatment effect independent of age. **A, B: Grip strength** declined significantly with age in all animals, regardless of treatment (p << 0.001); however rapamycin treatment affected males and females differently (treatment x sex, p = 0.003). Rapamycin-fed females had greater grip strength than controls at all ages measured; whereas grip strength in control and rapamycin treated males did not differ. Sample sizes varied, depending on age, control females, n = 17–7; rapamycin females, n = 27–4; control males, n = 22–4; rapamycin males, n = 31–3. **C, D: Stride length** increased in males and females until 27 months of age and then declined with increasing age (p << 0.001). Rapamycin treatment had no effect on stride length in either sex. Sample sizes varied, depending on age, control females, n = 15–6; rapamycin females, n = 21–5; control males, n = 19–4; rapamycin males, n = 26–9. **E, F: Rotarod performance,** measured as maximum latency to fall, was significantly affected by body mass (p << 0.001) and so body mass was included as a covariate in the analysis. With the effects of body size removed, females showed no effects of rapamycin treatment and males showed a marginally significant negative effect of rapamycin treatment on rotarod performance. The y-axis shows the residuals of rotarod performance (latency to fall) regressed against body mass. Sample sizes varied, depending on age, control females, n = 11–6; rapamycin females, n = 19–6; control males, n = 13–7; rapamycin males, n = 21–8.

As in humans, mouse grip strength declined with age in both male and female mice (Fig 5A and 5B, Age effect, *p* << 0.001). As with a number of other parameters, rapamycin-feeding had a sex-specific effect. Rapamycin-fed females’ grip was stronger than that of female controls at all ages measured (Treatment effect, *p* = 0.005), whereas grip strength of control and rapamycin-fed males did not differ (Treatment effect, *p* = 0.21). Although there were sex- and treatment-specific differences in body mass, these did not explain the grip strength results as body mass did not correlate with grip strength in either male (r^2^ = 0.07, *p* = 0.07) or female mice (r^2^ = 0.01, *p* = 0.90).

Stride length has previously been reported to decline with age in C57BL/6 mice [11, 42]. In both sexes stride length actually *increased* until 27 months of age, after which it declined with increasing age (Fig 5C and 5D, Age effect, *p* << 0.001). However, rapamycin treatment had no effect on age-related changes in stride length in either sex (Treatment X Age effect, females: *p* = 0.88, males: *p* = 0.48).

Rotarod performance in mice is well known to be strongly affected by body mass [11, 43, 44] as it was in this study (Mass effect, *p* < 0.001 for both sexes). Therefore, we used body mass as a covariate in our analysis. There was no clear pattern of change with age in either females (Fig 5E) or males (Fig 5F). Rapamycin treatment had no effect on female performance (Treatment effect, *p* = 0.42), but had a marginally significant negative effect on male rotarod performance (Treatment effect, *p* = 0.06). If body mass was ignored, there was still no rapamycin effect in females (Treatment effect, *p* = 0.65) and a significant negative effect in males (Treatment effect, *p* = 0.01).

### Cochlear Histology

**Fig 6:**
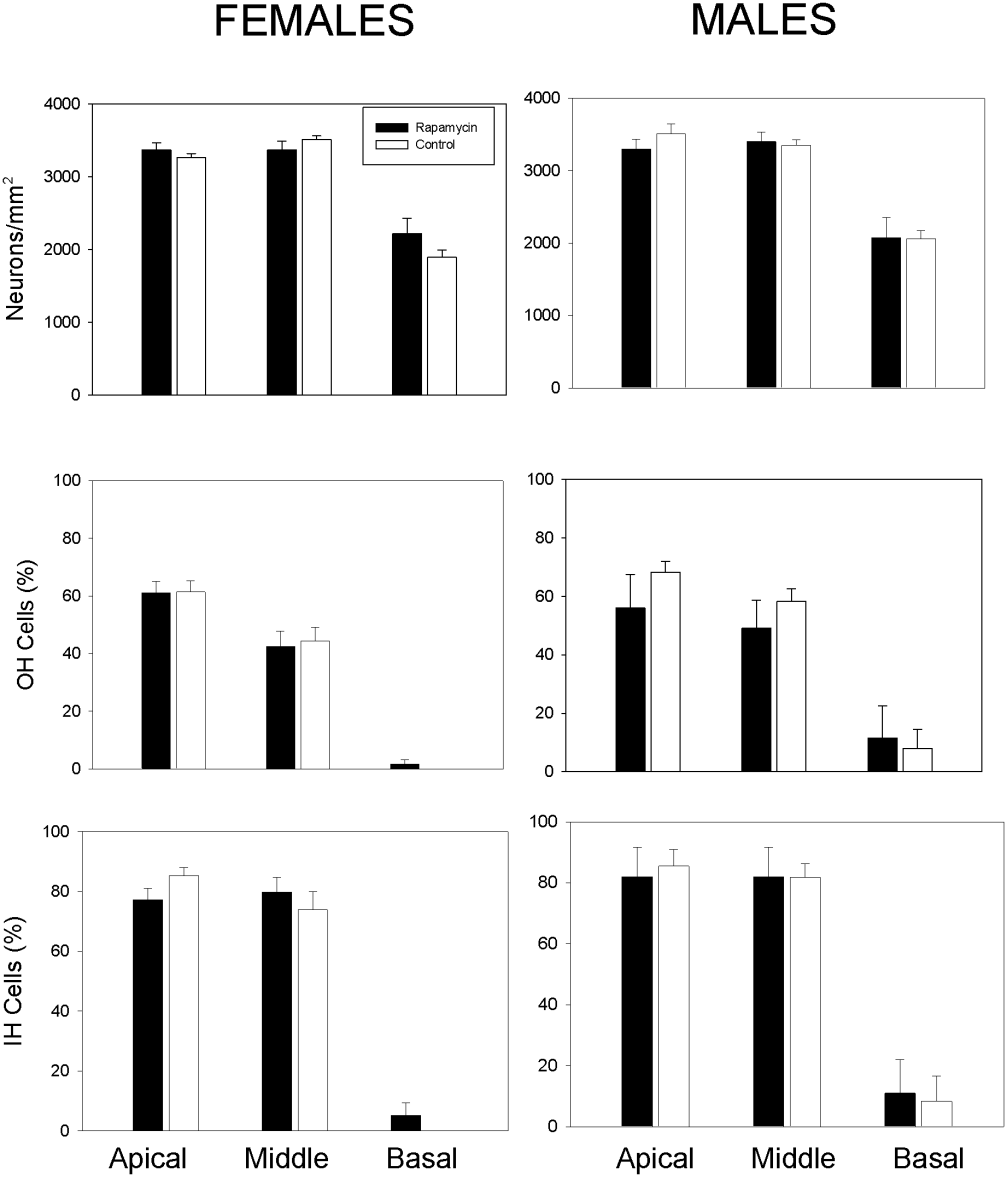
Age-related changes in inner ear histology was not altered by rapamycin treatment. **A, B: The number of cochlear neurons** in male and female mice were not statistically different between control and rapamycin-fed animals. **C,D: The number of outer hair cells** in male and female mice were not statistically different between control and rapamycin-fed animals. **E,F: The number of inner hair cells** in male and female mice were not statistically different between control and rapamycin-fed animals.

Age-related hearing loss is a common feature of aging and associated with loss of spiral ganglion neurons and sensory inner and outer hair cells in the cochlea. Cochlear histology in rapamycin-treated and control mice was assessed upon natural death or health-related euthanasia. There were no statistically significant differences in the ages of animals of either sex examined as a function of treatment (Mann-Whitney Rank Sum Test: males: *p* = 0.63, females: *p* = 0.45). Median ages examined were 24.4 and 25.4 months for control and rapamycin-fed males, respectively, and 24.5 and 25.5 months form control and rapamycin-fed females. No statistical differences between rapamycin-fed and control mice were observed (Fig 6). Collectively, rapamycin treatment had no impact on age-related cochlear pathology in males or females.

## Discussion

This study administered enteric rapamycin to C57BL/6J mice of both sexes and *longitudinally* monitored their phenotypes for a longer time—from 4 months of age until death—than any previous study. Therefore, individual, life-long trajectories of phenotypic change could be analyzed. Although survival was increased in both sexes [35], only some presumptive metrics of health were improved (female grip strength, reduced sleep fragmentation, body mass), a number showed no significant change, and at least one (male rotarod performance) was negatively affected. Age-related improvements in total sleep and greater sleep consolidation with rapamycin-feeding were observed in both sexes here, this is contrast to our previous OF study where only rapamycin treated males showed improvements in sleep [11]. These are the first reports of rapamycin affecting sleep.

At least 7 previous studies in an array of genotypes have reported enhanced longevity in mice administered rapamycin [2, 3, 5, 11, 15, 21, 45]. However, reports of rapamycin’s effects on health have been less numerous, none have made repeated assessments of the same animals longitudinally, and results have been considerably less uniform than the survival results. The health effects described here differ from those reported in our own previous study—performed in the same facility with the same equipment. Compared to our previous study, in which enteric rapamycin treatment was initiated at 19 months and continued throughout life (OF mice) [11], the YF mice in this study exhibited considerable differences in phenotype, and not just in the expected direction one might associate with earlier age and longer duration of rapamycin treatment (Table 1). For instance, we previously found rapamycin feeding significantly decreased mass-specific food consumption and resting metabolic rate in OF females relative to controls [11]. By contrast, YF females showed no difference in mass-specific food consumption and an *increased* resting metabolic rate compared to controls (Table 1). The pattern of age-related body mass and composition changes also differs between the two studies. In OF females, there was no significant difference in total body mass, percent body fat, or their rate of decline with age compared with controls; however YF females were significantly better than controls at maintaining body and fat mass into late life (Fig 2). Given its well-known role in growth and previous, shorter-term studies of its effects on body mass [3, 21, 45], the better maintenance of body and fat mass with age in our YF females was a substantial surprise. OF females showed no difference in fat-free mass compared with controls, whereas YF females had less fat-free mass than controls. Thus, the impact of rapamycin-feeding on various metabolic parameters *in females* appears to depend on the age of initiation and/or the duration of feeding in sometimes counterintuitive ways.

**Table 1:** Differences in two rapamycin studies.

C57BL/6 responses to rapamycin 14ppm				
Measure	Young-fed		Old-fed	
	Female	Male	Female	Male
Fat-free mass	↓***	↓*** §	---	↓* §
Total body mass	↑*** §	↓*** §	---	↑↓*** §
Percent fat	↑*** §	↓*** §	---	---
Mass-specific Food Consumption	---	↑** §	↓**	---
Light phase O_2_ consumption	---	---	↓*	---
Dark phase O_2_ consumption	↑**	---	---	---
Resting Metabolic Rate	↑*	↓*** §	↓**	---
Total activity (24h)	---	---	---	---
Total sleep	↑*	↑*	---	---
Sleep fragmentation	↓**	↓**	---	↓**
Grip strength	↑**	---	---	---
Rotarod performance	---	↓	↑*	↑*
Stride length	---	---	↑↓**	↑↓**
Survival	↑**	↑*	↑**	↑**

↑ Increased ↓ Decreased, ↑↓ Maintained with age,—No difference from controls

*p < 0.05

** p < 0.01

*** p < 0.001

^§^ Treatment X Age effect

Healthspan measures: young-fed measured between ages 16 and 29 months; old-fed measured at 25, 31 and 32 months.

Young-fed mice (rapamycin feeding begun at 4 mo, this study) compared with old-fed (rapamycin feeding begun at 19 mo) [11].

OF and YF male phenotypic changes also differed in response to rapamycin treatment, but mainly in easily understood directions. Several parameters such as percent body fat or mass-specific food consumption that did not achieve statistical significance in the shorter duration, OF study, did achieve significance in the longer, YF study. Unlike the current study, there was no statistically significant difference between the sexes in blood concentration of rapamycin in the OF study. However, both studies showed differences in the same direction, with females showing higher blood levels of rapamycin than males.

Sex-specific phenotypic effects of enteric rapamycin treatment in mice have been widely reported; in this study we report sex differences in the *direction* of phenotypic change. While males and females on enteric rapamycin were both significantly different from controls, they exhibited changes in the opposite direction in measures of body mass, percent fat and mass-specific resting metabolic rate. While this pattern of sex differences has not been previously reported, few phenotypic studies have been performed on both sexes (although see [15, 45]) and none have been performed longitudinally in both sexes over as long a time course as the present study. In the few phenotypic studies of both sexes that have been reported, it is not uncommon to find a statistically significant difference associated with enteric rapamycin treatment in one sex but not the other. Both this study and Miller, et al. [45] found significantly greater blood concentrations of rapamycin in females compared with males. In this study, the pattern was not a result of greater mass-specific food consumption in females, suggesting that such differences could be a consequence of greater bioavailability of rapamycin in females compared with males. Still, interpretation of differences in blood concentration should be treated with caution as they do not necessarily reflect tissue concentration of rapamycin [11, 35]. The mechanistic basis of these sex-specific differences certainly warrants further study.

A number of studies have previously reported health effects of rapamycin administration in shorter-term experiments. For instance, rapamycin treatment by injection in 22–24 month old male C57BL/6 mice improved several measures of health, including hematopoietic stem cell function [5] and 3 months of enteric rapamycin treatment in even older female C57BL/6 mice improved cardiac function and some metrics of behavioral, motor and skeletal phenotypes [1]. Similarly, chronically administered enteric rapamycin started at 9 months of age at a range of doses (4.7–42 ppm) maintained activity and blunted the expression of aging-related changes in heart, liver, adrenal glands, endometrial tissue and tendons in 20–22 month-old male and female UM-HET3 mice [15]. Likewise, chronic, enteric rapamycin treatment enhanced cognitive function in young C57BL/6 adult mice and reduced age-associated cognitive decline in older mice of both sexes [20] and Neff et al. [21] reported improved hepatic, immune and endocrine function in male mice of the same strain.

## Conclusions

In sum, longitudinal measures of health in C57BL/6J mice treated with enteric rapamycin (14 ppm) from 4 months of age revealed a number of previously unreported patterns. Although a number of health parameters were improved and a number unchanged, at least one (male rotarod performance) was marginally worse under rapamycin treatment. Other deleterious side effects including testicular atrophy, accelerated cataract formation, and glucose insensitivity have been reported in shorter-term (~1 year) rapamycin feeding studies [15, 45]. Whether some of these effects, such as glucose insensitivity abate with longer-term treatment remains an intriguing question. We also observed considerable sex-specificity in the effects of enteric rapamycin treatment, including not only significant effects in one sex that were absent from the other, but also opposite phenotypic effects between the sexes. This phenomenon may provide insights into mechanisms underlying sex differences in aging and deserves further inquiry.
